# T-*6b* allocates more assimilation product for oil synthesis and less for polysaccharide synthesis during the seed development of *Arabidopsis thaliana*

**DOI:** 10.1186/s13068-017-0706-3

**Published:** 2017-01-21

**Authors:** Yunkai Jin, Jia Hu, Xun Liu, Ying Ruan, Chuanxin Sun, Chunlin Liu

**Affiliations:** 1 Hunan Provincial Key Laboratory of Crop Germplasm Innovation and Utilization, Hunan Agricultural University, Changsha, 410128 China; 2 Department of Plant Biology, Uppsala BioCenter, Linnean Center for Plant Biology, Swedish University of Agricultural Sciences, PO Box 7080, SE-75007 Uppsala, Sweden; 3 Key Laboratory of Education, Department of Hunan Province on Plant Genetics and Molecular Biology, College of Bioscience and Biotechnology, Hunan Agricultural University, Changsha, 410128 China

**Keywords:** T-*6b*, Endosperm expression, Starch, Mucilage, Lipid, Biosynthesis

## Abstract

**Background:**

As an *Agrobacterium tumefaciens* T-DNA oncogene, T-*6b* induces the development of tumors and the enation syndrome in vegetative tissues of transgenic plants. Most of these effects are related to increases in soluble sugar contents. To verify the potential roles of T-6b in the distribution of carbon in developing seeds, not in vegetative tissues, we fused an endosperm-specific promoter to the T-*6b* gene for expression in transgenic *Arabidopsis thaliana* plants.

**Results:**

The expression of T-*6b* in reproductive organs did not induce the development of the enation syndrome, and moreover, promoted endosperm expansion, which increased the total seed biomass by more than 10%. Additionally, T-6b also increased oil content in mature seeds by more than 10% accompanied with the decrease of starch and mucilage content at the same time.

**Conclusions:**

T-6b enhances seed biomass and helps oil biosynthesis but not polysaccharides in reproductive organs without disturbing vegetative growth and development. Our findings suggest T-*6b* may be very useful for increasing oil production in biodiesel plants.

## Background

Many *Agrobacterium tumefaciens* T-DNA genes belong to a family of the so-called developmental plasticity genes [1]. This family includes the genes *3′*, *5*, *6a*, *6b*, *rolB*, *rolC*, *orf8*, *orf13*, *lso*, and various other T-DNA genes [2], which can induce the formation of tumors, known as crown galls, on many dicotyledonous plants. The *6b* gene is located at the *tml* locus [3], and has been detected among the T-DNA of all *A. tumefaciens* strains. This gene appears to affect the proliferation of plant cells. Hooykaas et al. [4] were the first to report that a *6b* gene (i.e., A-*6b* from strain Ach5) induces the production of tumors on *Nicotiana glauca* and *Kalanchoe tubiflora* stems. Therefore, this gene was confirmed as an oncogene. The *6b* genes were subsequently determined to be responsible for the development of tumors in *Nicotiana tabacum* plants [5, 6]. Variants of the 6b protein differ regarding their ability to induce tumor development, with T-6b having the strongest oncogenic activity. The following four amino acid fragments are necessary for T-6b to be able to produce tumors in infected plants (the sequences and coordinates for T-6b are given in parentheses): L.YVY. (LVYVYL) at position 122–127 (62–67),…AT (GTVVT) at position 151–155 (89–93), .PPY (IPPW) at position 157–160 (95–98), and F.AI (FLAI) at position 196–199 (131–134). The proline residue at position 159 (97) is the only residue that is conserved in all RolB-like proteins. Additionally, the LTG sequence at position 57–59 in the T-6b protein is a determinant for the production of large tumors [2]. The results presented herein demonstrate that the *6b* coding sequences contribute to the differences in tumor development among the oncogenes. However, the *6b* promoters may also influence tumor formation.

The ectopic expression of *6b* genes in plants results in various symptoms, including enations, catacorollas, tubular leaves, expanded cotyledons, corkscrew stems, short and thick roots, ectopic shoots on hypocotyls, fragmented leaf primordia, vein thickening, hyponastic petioles, and epigastric leaf veins. This unique set of 6b-induced modifications is called the enation syndrome [7, 8], which is the consequence of localized *6b* expression and the movement of one or more unidentified 6b-induced enation factors. These factors are transported through the phloem, cross graft junctions, and mainly affect the growth of young tissues [7, 9]. Tumors form in plants expressing T-*6b* mainly because of increasing osmolality due to glucose and fructose. Leaves expressing T-*6b* contain considerably more glucose, fructose, and sucrose than mock-infiltrated leaves [10]. Glucose and fructose contents increase in parallel by a factor of 3 or 4, while sucrose levels increase by a factor of 2 or 3 [10]. Early studies showed that the phytohormone-like effects of 6b to enhance cell expansion and epigastric growth had no relationship with auxin and cytokinin [7, 9]. However, Takahashi et al. [11] recently observed abnormal auxin and cytokinin accumulation in dex-AK-6b seedlings, indicating that these hormones are important for the 6b phenotype.

As photosynthates, glucose and sucrose are substrates used to synthesize organic polymers. Starch and oil are the two most important seed storage polymers, and they are produced in two typical pathways that require glucose or sucrose [12]. In monocots, starch is present mainly in the endosperm, while oil mainly accumulates in the embryo. In almost all dicots, seed development includes an endosperm phase, during which starch accumulates, and a cotyledon phase, in which oil accumulates [13–15]. Starch biosynthesis in dicots temporarily increases the local sink strength to form a carbohydrate reserve that can be used for metabolic or growth processes [16]. Although starch is present during the early seed developmental stage of dicotyledonous plants, lipid is used as the final storage material in wild-type seeds [15, 17]. Oilseeds accumulate lipids as a source of energy and carbon for seedling growth following germination [18–20]. Triacylglycerols (TAGs) are the major storage lipids that accumulate in developing seeds, flower petals, pollen grains, and fruits of several plant species [21]. In Arabidopsis seeds, TAGs are mainly stored in embryo, but the endosperm also accumulates 10–15% of seed oil [22, 23]. The proanthocyanidin and mucilage contents, which are positively controlled by GLABRA2, are negatively correlated with the amount of TAGs in the seed [24–26]. Plant TAGs are important nutrient sources for humans. They also serve as precursors for the industrial production of chemicals and as renewable biofuels because they consist of abundant forms of reduced carbon that are highly enriched in energy [27–29]. Because of the importance of TAG, to improve the seed oil content has become an important target in the field of plant research.

Starch degradation and fatty acid formation in dicot seeds are related to the carbon flux from carbohydrates to oils, and the underlying mechanism has been the focus of several studies [30–33]. Modified vegetative growth induced by 6b occurs because of changes to the distribution of carbohydrates [2, 9, 10]. We hypothesized that the expression of T-*6b* during *Arabidopsis thaliana* endosperm development affects the allocation of carbon and results in increased availability of soluble sugars for developing seeds. To test this hypothesis, we transformed *A. thaliana* plants with T-*6b* for endosperm-specific expression. We observed that T-*6b* expression led to increase lipid synthesis but decrease in the production of starch and mucilage and accelerated starch and sucrose degradation. Additionally, *A. thaliana* seed yields increased, with no effects on seed phenotype. These results confirm that T-6b influences carbon allocation in reproductive tissues in the absence of the enation syndrome, which is particularly relevant for the metabolic engineering of biodiesel plants for increased seed oil production.

## Methods

### Plant growth and seed biomass analysis

*Arabidopsis thaliana* plants were grown in a phytotron at 22 °C with 70% relative humidity under a 16-h light/8-h dark cycle (light intensity: 250 µmol photons m^−2^ s^−1^). The Col-0 ecotype was used as the wild-type control. For 100-seed weight measurements, seeds were harvested from six plants and dried at 37 °C for 2 days. The following harvested seeds were also dried using the same method. The seeds collected from six plants underwent sample quartering, after which 100 seeds were randomly selected from each sample. To determine the seed weight per silique, 10 mature siliques were collected from the same position on the main stem of every third wild-type and transgenic plant. The harvested seeds were dried and then weighed. The seed weight measurements were completed using six replicates. The number of seeds per silique was calculated using siliques collected from the same position of wild-type and transgenic plants at 12 DAF. Seeds were counted using a stereomicroscope, and 10 replicates were analyzed. To determine seed yield per plant, seeds from six randomly selected plants were dried and weighed to calculate the average seed weight per plant.

### Plasmid construction and plant transformation

The general molecular cloning procedures were performed according to previously developed protocols [34]. A fragment spanning nucleotides 9013–9636 of T-*6b* gene of the *A. tumefaciens* sequence corresponding to GenBank accession number X56185 was fused to the promoter of the rice glutelin precursor gene (*Gt1*p: nucleotides 1–1274; GenBank accession number AY585231). The fused DNA fragment was cloned into the pFGC5941 vector for subsequent transformation of *A. thaliana*. Plant transformations were mediated by *A. tumefaciens* strains GV3101. The floral dip method was used to transform *A. thaliana* plants [35, 36]. Basta (Duchefa, Haarlem, the Netherlands) was added to the 1/2 MS medium used to screen transformed *A. thaliana* plants. Transgenic plants were analyzed by PCR using primers 6bF and 6bR (Table 1).

**Table 1:** Primer details

Primer name	Gene name	Primer sequence (5′-3′)
Primers for semi-quantitative/quantitative PCR		
QIKU1F	*IKU1*	AGTTTTGGTCTAATACAGCTGAG
Q-IKU1R		GGTTGAGACTGAGACTGAGATT
Q-IKU2F	*IKU2*	CGTGTGAGACAAGCGTTAGC
Q-IKU2R		GAGGAGACTTGTCCGTGCAT
Q-A/N-InvAF	*A/N*-*InvA*	CATACATTACAGCTTCAGAGTTGGG
Q-A/N-InvAR		GAGAATAATCCACCACAAACCAGAA
QSBE2.1F	*SBE2.1*	ATCATGGACTGCAGGTCGAAT
QSBE2.1R		TCCCGCTAACATCTTCGCCG
QSUS2F	*Sus2*	AAGCAAGAACAATGTTGGGCA
QSUS2R		GCTCAGTAAACCAACATGCTCATC
QAPS1F	*APS1*	TTCCTGATTTTAGTTTCTATGACCG
QAPS1R		TGATGAATTTTGCAGTTCTTGATAA
QWRIF	*WRI1*	AATTTTCCGGCAGAGACGTACA
QWRIR		CCTCCTGCGTATTATAGGTGCC
QGL2F	*GLABRA2*	GAAGCTCGTCGGCATGAGTGGG
QGL2R		TCTCTCGATTTCACTGTCTGGATTG
QDGAT1F	*DGAT1*	TTTGGTTAAACATATTGGCAGAGC
QDGAT1R		AATGATAATGGCGAGTGTCTTTGGT
Q-MINI3F	*MINI3*	TTTGATGATATTGCAACGGAA
Q-MINI3R		GATCCTTTGTGTCTTGCTTGT
Actin2F	*Actin2*	ATGGCTGAGGCTGATGATATTCAAC
Actin2R		TCTCAGCACCAATCGTGATGACTTG
QUBQ10F	*Ubiquitin10*	AGGTACAGCGAGAGAAAGTAGCA
Q-UBQ10R		TAGGCATAGCGGCGAGGCGT
Q6bF	T-*6b*	GACGAGATCAACGGTGCAAG
Q6bR		TGACAAGGTCTCCGAACTGG
Primers used in the construct		
Gt1F	*Gt1 promoter*	GGAATTCCAGGTCATAGGGAGAGGGAGCTTTTG
Gt1R		TTGGCGCGCCAAGTTGTTGTAGGACTAATGAACTGAA
6bF	T-*6b*	TTGGCGCGCCAAATGACGGTAGCCAATTGGCAGGTTC
6bR		CCTTAATTAAGGCTATGCCGAAAGACGGCTTGACCCT

### Light microscopy

Mature *A. thaliana* seeds and seeds stained with iodine or ruthenium red solutions were observed using the SMZ1000 stereomicroscope (Nikon) at different magnifications.

### Semi-quantitative and quantitative PCR

Seeds, siliques, or leaves were ground to a fine powder in liquid nitrogen, after which total RNA was isolated using TRIzol reagent (Invitrogen, Carlsbad, CA, USA) according to the manufacturer’s instructions. Samples were treated with DNase I to remove any residual genomic DNA. Total RNA (1 µg) was used to synthesize cDNA with the RevertAid™ First Strand cDNA Synthesis Kit (Fermentas, USA). Semi-quantitative PCR was conducted using equal amounts of synthesized cDNA (i.e., based on the signal of reference gene products). The synthesized cDNA concentration was adjusted to 5 ng µL^−1^, and 15 ng was used for qPCR analysis. The qPCR was completed in a 20-µL solution containing 5 µM specific primers and a SYBR Green PCR Master Mix (Fermentas). The qPCR program was as follows: 95 °C for 4 min; 40 cycles of 95 °C for 30 s and 60 °C for 30 s. The melting curve analysis was completed by increasing the temperature from 60 to 95 °C at a rate of 0.05 °C s^−1^. The specificity of the qPCR amplifications was verified by the presence of a single band following gel electrophoresis. Relative expression levels were calculated using the comparative *C*_t_ method [37], with gene expression levels normalized against the values of the *Ubiquitin10* housekeeping gene. Data were analyzed using the Student’s *t* test.

### Determination of carbohydrate content and starch staining

Starch, sucrose, and glucose were extracted and analyzed as described by Sun et al. [38] and Zhang et al. [39] and according to the protocols provided in CFHN-K-SUCGL and CFHN-K-TSTA Megazyme kits (Bray, Co. Wicklow, Ireland). For starch staining, *A. thaliana* seeds were cut with a surgical blade and incubated in a 50% (v/v) iodine solution (i.e., Lugol’s solution) for 20 min [39]. Excess staining solution was removed by washing seeds with distilled water.

### Protein isolation and enzyme activity analysis

Siliques were collected at 7 DAF, frozen in liquid nitrogen, and ground to a fine powder in mortars. Total protein was extracted from 100 mg powder using the Plant Total Protein Extraction Kit (Sigma-Aldrich, St. Louis, MO, USA) according to the manufacturer’s recommended procedure, but without protease inhibitors. The extracted proteins were separated by polyacrylamide gel electrophoresis (150 V for 1.5–2 h) using a 4–12% gradient gel. The separated proteins were visualized using Coomassie blue to estimate protein abundance [37, 40]. Equal amounts of protein from different samples were added to 100 mg mL^−1^ starch solutions and incubated at 28 °C for 4 h. The starch-degraded solutions were stained with iodine to analyze the remaining starch granules.

### Lipid analysis

100 mg seeds were used for lipid analysis for whole seed samples and 3 mg seeds were taken for the lipid analysis of separated endosperm and embryo. The separation of embryo and endosperm was carried out according to the method as described by Penfield et al. [22]. The TLC and GC experiments were completed using modified versions of published procedures [41–43]. Briefly, seed, endosperm, or embryo was homogenized in a Potter-Elvehjem homogenizer with homogenate solution [1 mL 0.15 M acetic acid and 3.75 mL methanol:chloroform (2:1; v/v)]. The extract was transfered to a glass tube (with a screw cap) and combined with 1.25 mL of chloroform from rinsing of the homogenizer. H_2_O of 1.25 mL was added to the extract. The sample was mixed vigorously and centrifuged (approximately 250 rcf for 2 min). The chloroform phase was carefully transferred to a new glass tube. Extraction volumes equivalent of 2.5 mg seed mass for whole seed sample, 0.3 mg for embryo, and 0.6 mg for endosperm were taken for TLC analysis. Extraction volumes equivalent of 0.5 mg seed mass for the three samples were used for GC analysis. After dried under nitrogen flow, lipids were re-dissolved with 40 µL hexane. Then samples were loaded onto TLC Silica Gel 60 plates (Merck) and separated with a hexane:diethyl ether:acetic acid (70:30:1, v/v/v) solution. Plates were sprayed with 20% phosphomolybdic acid hydrate in ethanol to visualize lipids or sprayed with 0.05% primuline for further GC analysis. Relative intensity of different lipid species was analysis by the software ImagJ [44, 45]. The TAG spots on the TLC plates were transferred to glass tubes (with screw caps) and methylated with 2 mL 2% (v/v) H_2_SO_4_ in methanol at 90 °C for 90 min. Hexane (2 mL) and H_2_O (3 mL) were added to the solutions, which were then vigorously mixed for 20 s. The fatty acid methyl esters in hexane were analyzed by GC, which was completed using a 6890 N gas chromatograph (Agilent Technologies, Wilmington, DE, USA) equipped with an auto-sampler (CTC Analytics AG, Zwingen, Switzerland) and a flame ionization detector. The data were analyzed using the ChemStation (revision B.02.01) program (Agilent Technologies).

### Mucilage analysis

Seed coat mucilage was stained with 0.01% ruthenium red under different conditions to produce optimal results [46, 47]. Mature dried seeds and seeds imbibed in water for 1 h were stained directly to analyze the mucilage biomass at the epidermal cells of *A. thaliana* seed coats.

### Primers

The details of the primers used in this study are listed in Table 1. The oligonucleotides were purchased from Genscript (Nanjing, China).

## Results

### T-*6b* expression in the endosperm increases seed biomass

**Fig. 1:**
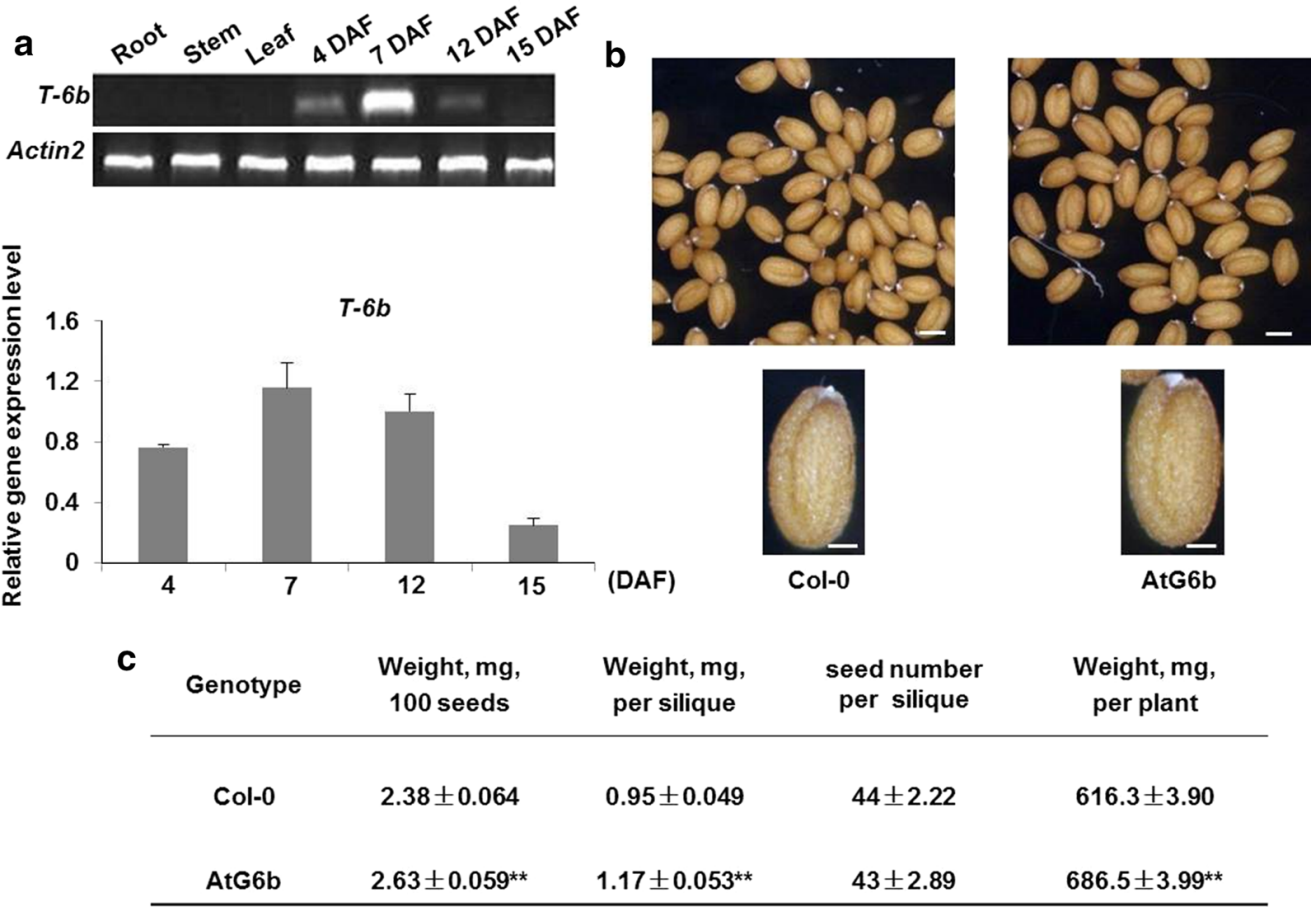
Expression of *Gt1*p:T-*6b* in *Arabidopsis thaliana* leads to biomass changes. **a** Expression profile of *Gt1*p:T-*6b* in *Arabidopsis thaliana* based on semi-quantitative PCR (*upper panel*) and quantitative PCR (*lower panel*). DAF, days after flowering. *Error bars* represent standard deviation and *n* = 3. **b** Comparison of seed size between Col-0 and AtG6b plants. *Bars* in *upper* and *lower panels* = 300 and 100 µm, respectively, *n* > 6. **c** Characteristics of Col-0 and AtG6b plants. Biomass indices include 100-seed weight, seed weight per silique, and seed yield per plant. *P < 0.05 and **P < 0.01 by Student’s *t*-test. Values are presented as the mean ± standard deviation and *n* = 3

The promoter of the glutelin precursor gene (*Gt1*), which is primarily expressed in the rice endosperm [48], was fused to T-*6b* for subsequent expression in transgenic *A. thaliana* (AtG6b) plants. As expected, T-*6b* was expressed in developing seeds, but not in vegetative tissues. Its transcription level sharply increased from 4 to 7 days after flowering (DAF), which corresponds to the starch synthesis stage in *A. thaliana* plants. The T-*6b* transcript abundance was the highest at 7 DAF, and then gradually decreased until transcripts were undetectable after 12 DAF (Fig. 1a), which coincides with the end of the endosperm development stage [15].

**Fig. 2:**
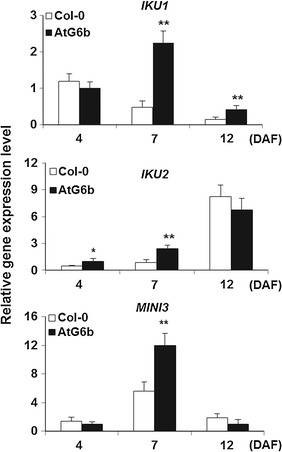
Transcript levels of *IKU1*, *IKU2,* and *MINI3* in AtG6b plants. Gene expression levels were analyzed in developing siliques at 4, 7, and 12 days after flowering. *Error bars* indicate the standard deviation and *n* = 3. *P < 0.05 and **P < 0.01 by Student’s *t*-test

Seed biomass was determined according to 100-seed weight, seed weight per silique, seed number per silique, and seed yield per plant. Significant differences were observed between wild-type Columbia (Col-0) and AtG6b plants for all indices, except for seed number per silique. Additionally, the enation syndrome was not detected in any seed (Fig. 1b, c). *IKU1*(*HAIKU1*, AT2G35230), *IKU2*(*HAIKU2*, AT3G19700), and *MINI3*(*MINISEED 3,* AT1G55600) are responsible for endosperm development and their overexpression usually leads to increased seed size [49]. We analyzed the expression levels of these three genes by quantitative polymerase chain reaction (qPCR). The *IKU1*, *IKU2*, and *MINI3* transcript levels during the early stages of silique development were higher in AtG6b plants than in wild-type plants, especially at 7 DAF (Fig. 2). These results suggest that T-6b increases seed biomass by up-regulating the expression of *IKU1*, *IKU2*, and *MINI3*.

### T-6b regulates carbohydrate metabolism in developing seeds

**Fig. 3:**
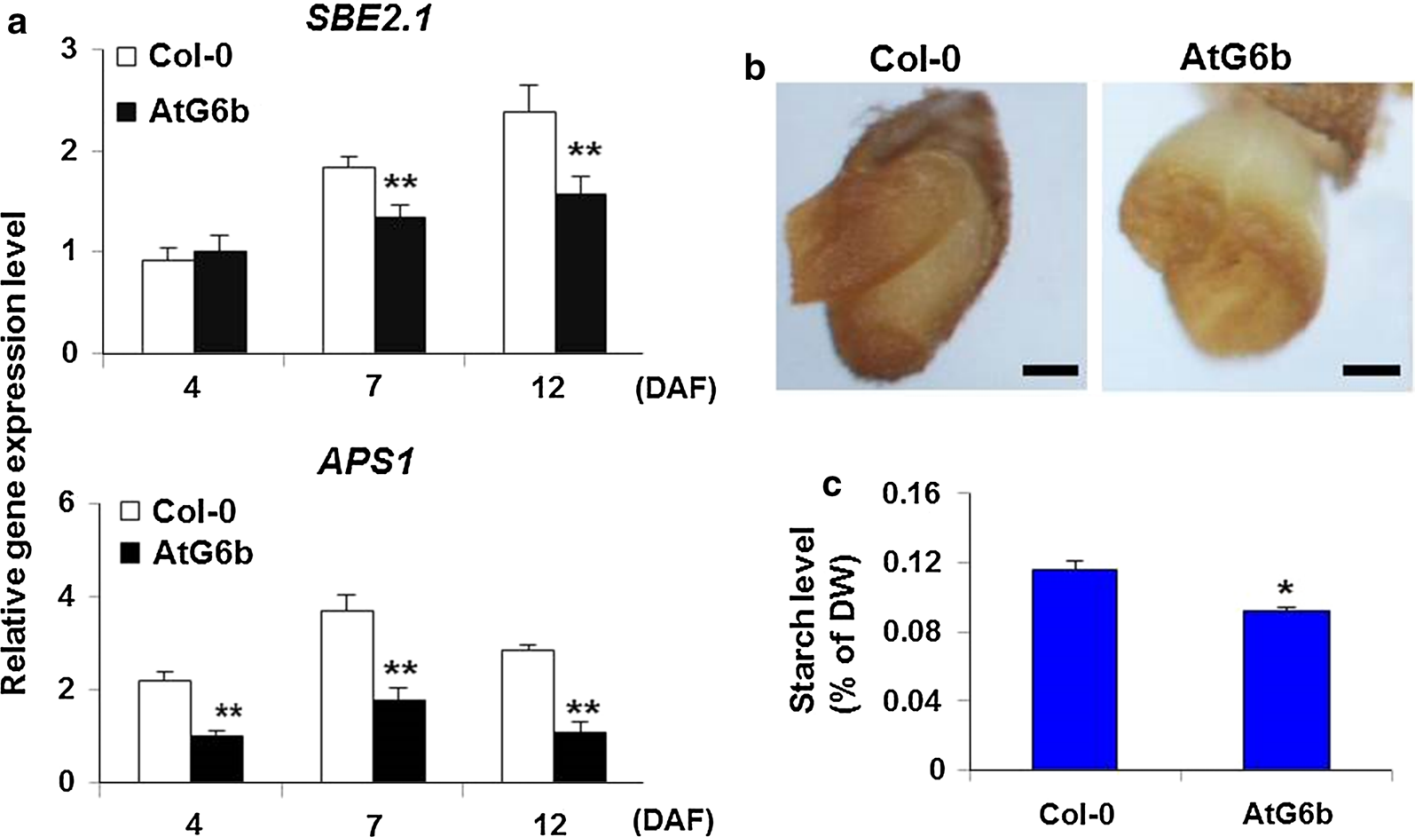
Changes in starch levels and relative gene expression levels. **a** Expression levels of *BE2.1* and *APS1* genes influencing starch content in *Arabidopsis thaliana* siliques at different developmental stages. Error bars represent standard deviation and *n* = 3. **P < 0.01 by Student’s *t*-test. **b** Cut mature seed and stained with iodine solution. *Bar* = 100 µm. **c** Changes in the starch contents of *Arabidopsis thaliana* seeds. *Error bars* indicate the standard deviation and *n* = 3. *P < 0.05 by Student’s *t*-test

To investigate the status of starch synthesis, we first analyzed the expression levels of *SBE2.1* (Starch Branching Enzyme 2.1, EC.2.4.1.18, AT2G36390) which is responsible for the starch branching, and *APS1* (ADP Glucose Pyrophosphorylase 1, EC.2.7.7.27, AT5G48300) encoding the small subunit of AGPase (ADP glucose pyrophosphorylase) that is crucial important for the initial of starch synthesis [50]. The qPCR results revealed that the *SBE2.1* and *APS1* transcript levels were considerably lower in AtG6b plants than in Col-0 plants at 4, 7, and 12 DAF (Fig. 3a). In mature seeds, starch was stained with an iodine solution. The intensity of the brown stain was lower in testa-peeled AtG6b seeds than in Col-0 seeds (Fig. 3b). Further analyses indicated that starch contents were significantly (*P* < 0.05) lower in AtG6b seeds than in Col-0 seeds (Fig. 3c). The results suggest that T-6b can limit starch synthesis.

**Fig. 4:**
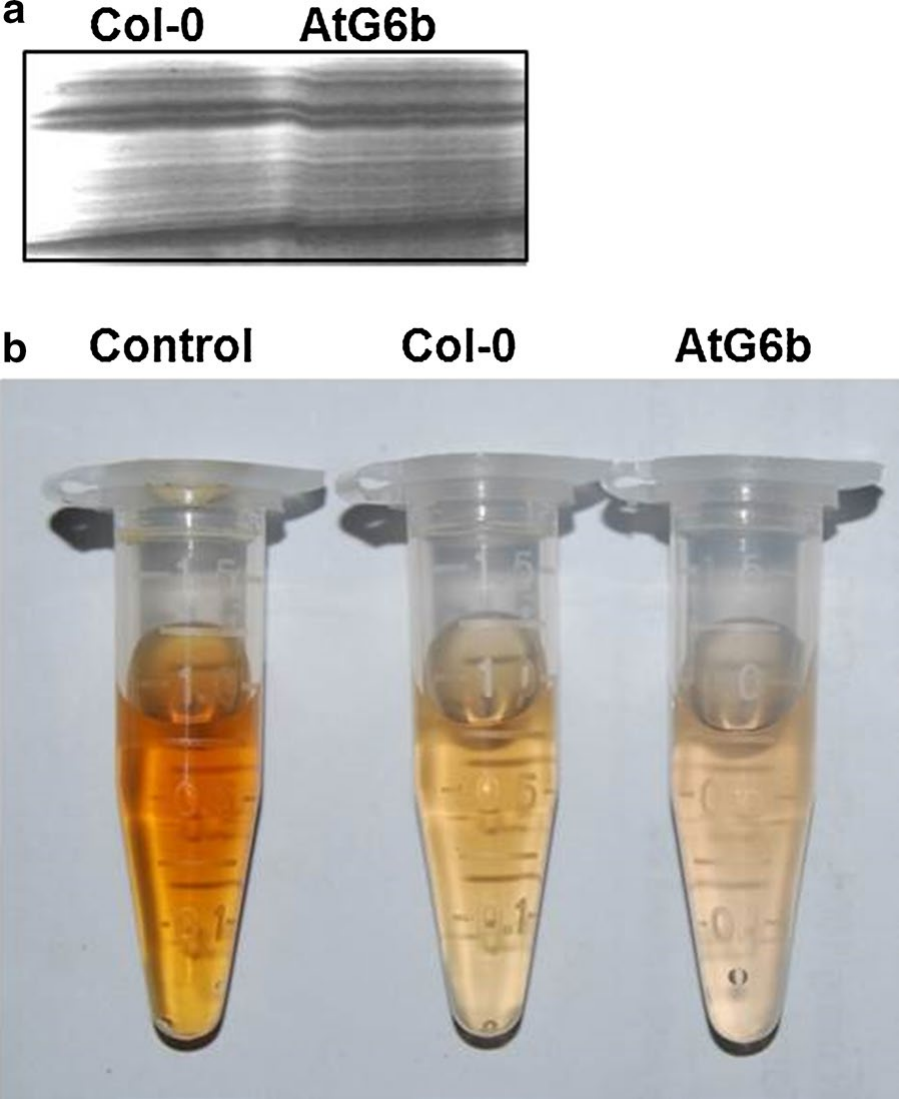
Starch degradation analysis.** a** Coomassie blue-stained total proteins isolated from siliques at 7 days after flowering. **b** Total starch-degrading enzyme activity. Proteins extracted from siliques at 7 days after flowering were incubated with starch standard solutions. Samples were then stained with an iodine solution. An incubation without protein was used as a control

Starch synthesis and degradation occur concurrently in developing seeds [15, 51], resulting in fluctuating starch contents. To confirm the effects of T-6b on starch metabolism, in vitro biochemical detection experiments were conducted. Total proteins were extracted from siliques at 7 DAF and stained with Coomassie blue following gel electrophoresis (Fig. 4a). Equal amounts of total protein were then used to degrade starch. The starch samples degraded by proteins isolated from AtG6b siliques produced less intense iodine staining compared with the starch samples treated with the proteins extracted from wild-type siliques (Fig. 4b). The results indicate that T-6b decreases starch content in *A. thaliana* seeds by inhibiting synthesis and accelerating degradation.

**Fig. 5:**
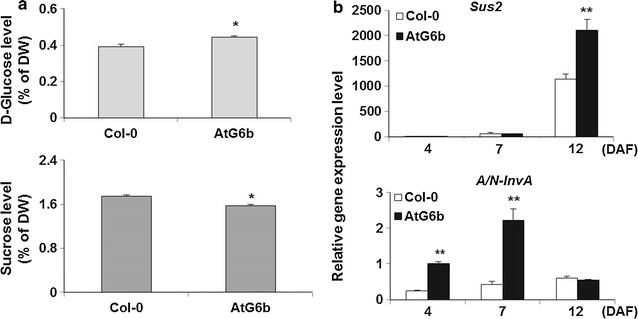
Glucose and sucrose responses to the expression of T-*6b* in seeds.** a** d-glucose and sucrose levels in mature seeds. **b** Transcription levels of *Sus2* and *A/N*-*InvA* genes responsible for sucrose degradation. *Error bars* indicate the standard deviation and *n* = 3. *P < 0.05 and **P < 0.01 by Student’s *t*-test

A previous study concluded that the expression of T-*6b* in tobacco leaves can increase soluble sugar contents [10]. Therefore, we speculated that the soluble sugar contents of AtG6b seeds were also influenced by T-*6b* expression. To prove this hypothesis, we investigated the glucose and sucrose contents in mature *A. thaliana* seeds. We observed that the glucose content was higher in AtG6b seeds than in Col-0 seeds, while sucrose abundance exhibited the opposite pattern (Fig. 5a). It is possible that the lower sucrose level in AtG6b seeds was caused by increased sucrose degradation. We next investigated the expression levels of *Sus2* (Sucrose Synthase 2, EC.2.4.1.13, AT5G49190) which is an isoform of sucrose synthase mainly expressed at the later seed development stage responsible for the sucrose degradation, and *A/N*-*InvA* (Alkaline/Neutral Invertase A, EC.3.2.1.26, AT1G56560), encoding a kind of neutral invertase [52, 53]. As expected, the expression levels of these two genes were higher in AtG6b plants than in wild-type controls (Fig. 5b). The findings suggest that T-*6b* expressed in endosperm of *A. thaliana* seeds can increase glucose contents and promote sucrose degradation.

### T-6b promotes the biosynthesis of oil in seeds

**Fig. 6:**
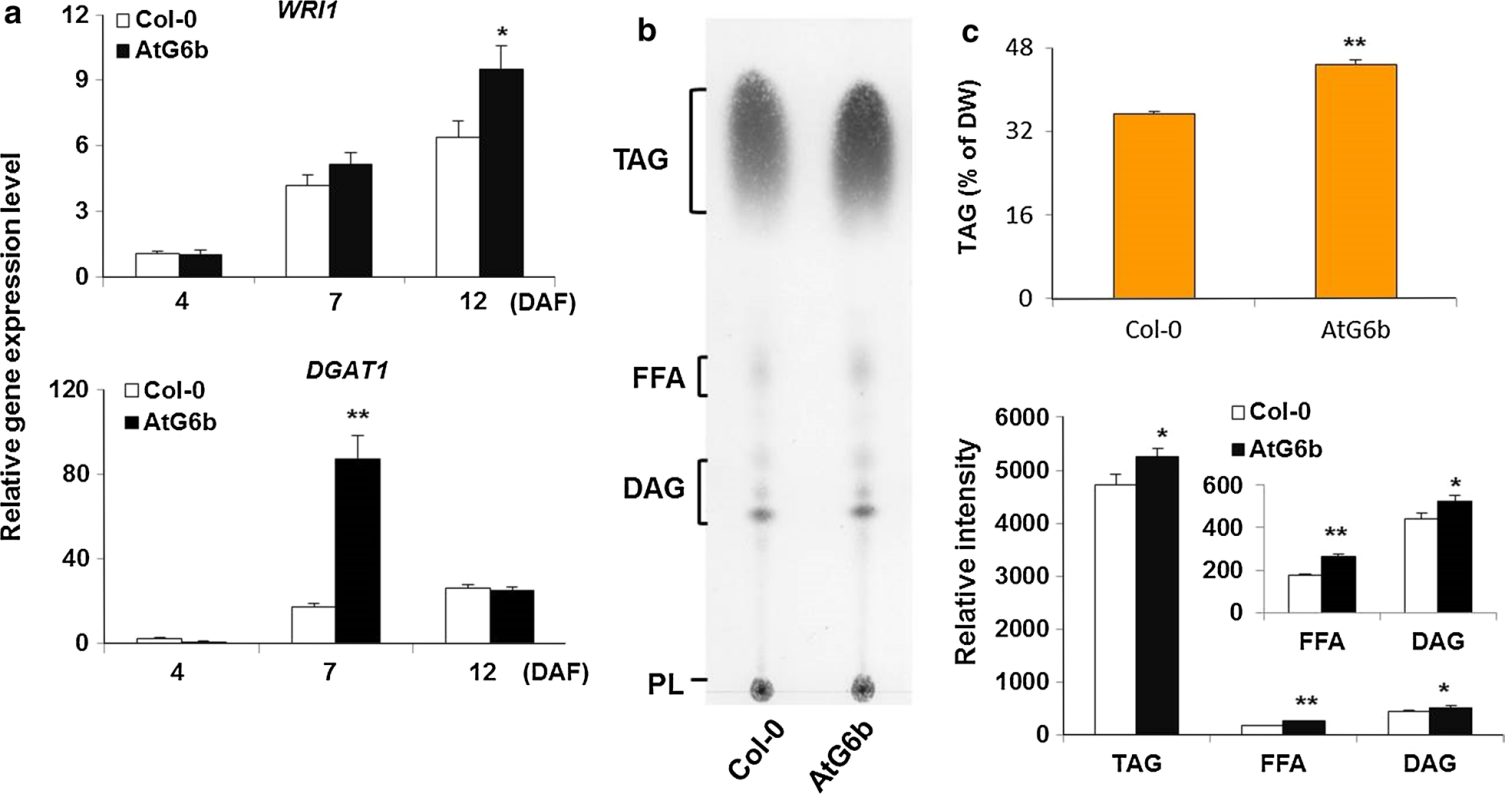
Role of T-6b during fatty acid metabolism. **a** Expression levels of *WRI1* and *DGAT1* genes in siliques at different developmental stages. **b** Thin layer chromatography analysis of lipids isolated from mature seeds. Loading volume equals 2.5 mg mature seeds for both Col-0 and AtG6b. **c** Total TAG quantities in mature seeds of wild-type and AtG6b were determined by gas chromatography (*up panel*). Relative intensity of lipid species was calculated by ImagJ (*below panel*). *P < 0.05 and **P < 0.01 by Student’s *t*-test. *Error bars* indicate the standard deviation and *n* = 3

Starch in endosperm is considered “transitory starch” in oilseeds, and its degradation is accompanied by the formation of fatty acids in cotyledons [51]. We speculated that the role of T-6b in the carbohydrates metabolism has a direct or indirect effect on lipid synthesis. Siliques at different developmental stages were collected for gene expression analyses, while mature seeds were harvested for lipid content investigations. We first determined the *WRI1* (WRINKLED1, AT3G54320) and *DGAT1* (Diacylglycerol Acyltransferase 1, EC.2.3.1.20, AT2G19450) expression levels [54–60]. Expressing T-*6b* during endosperm development stimulated the transcription of *WRI1* and *DGAT1*. The *WRI1* expression level increased at 7 and 12 DAF, while *DGAT1* transcription sharply increased at 7 DAF (Fig. 6a).

A lipid content assay was conducted using mature *A. thaliana* seeds. Based on thin layer chromatography (TLC) results, we determined that the TAG spot for AtG6b seeds was larger than the corresponding spot for Col-0 seeds (Fig. 6b). Furthermore, quantitative analyses by gas chromatography (GC) revealed that the TAG content in dry AtG6b seeds (44.72% ± 0.89) was approximately 10% higher than the TAG content in dry Col-0 seeds (35.41% ± 0.34) (Fig. 6c). In generally, up-regulated expression of *WRI1* leads to the increase in the levels of fatty acid (FA) [55, 56]. By comparing the corresponding grayscale of different lipid species from Col-0 and AtG6b on TLC plate by ImagJ, the relative intensities of AtG6b seed lipid bands were significantly higher than the ones of Col-0 seed lipid bands (Fig. 6c), indicating that FA content in transgenic seeds significantly higher than the one in wild-type seeds.

**Fig. 7:**
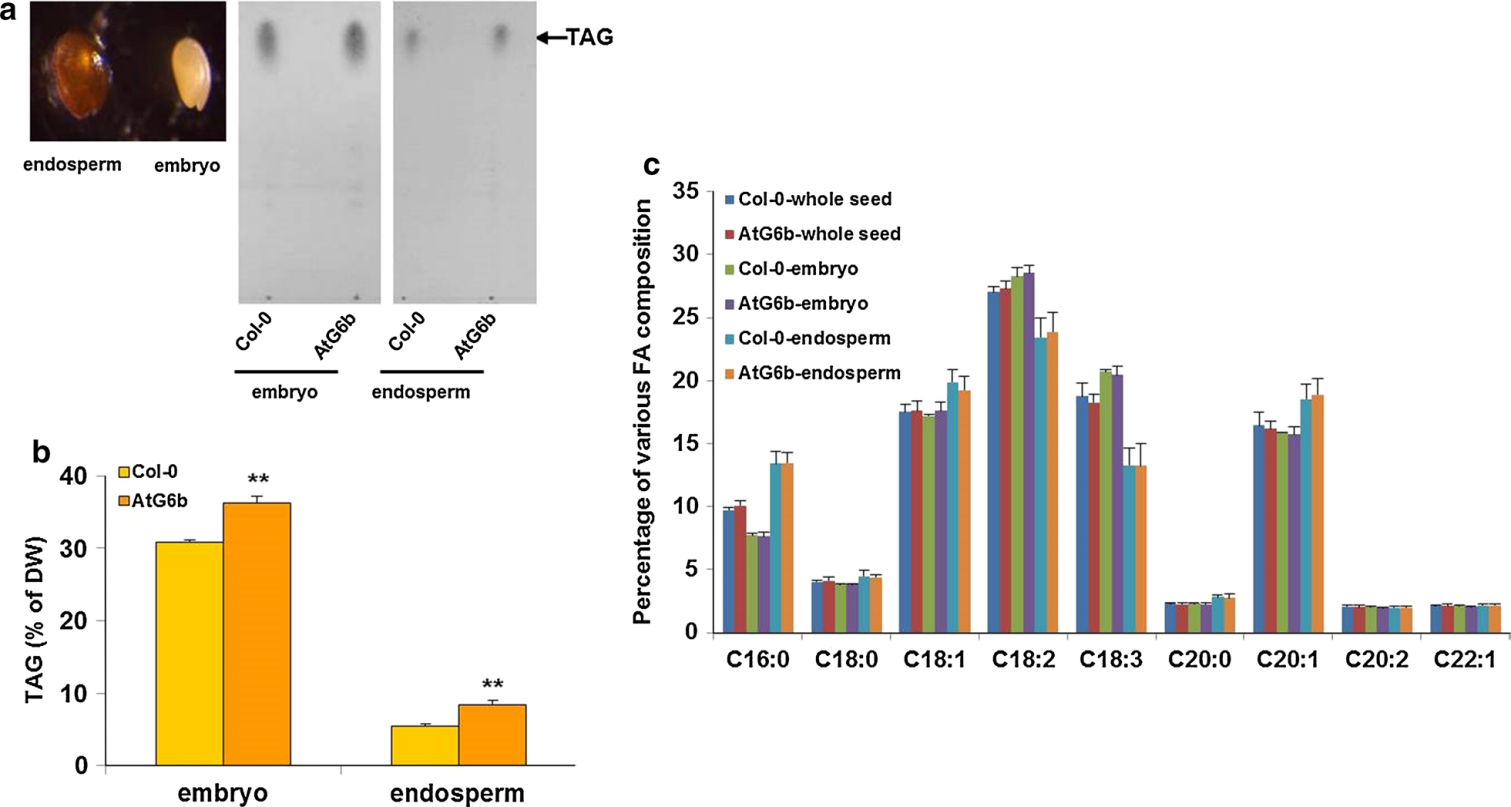
Lipid content and fatty acid composition analysis in both embryo and endosperm. **a** The dissected endosperm/seed coat and embryo of mature Arabidopsis seeds after soften in water for 6 h at 4 °C (*left panel*). TLC separation of lipids isolated from endosperm (extraction volumes equivalent to 0.6 mg seed mass for both Col-0 and AtG6b) and embryo (extraction volumes equivalent to 0.3 mg seed mass for both Col-0 and AtG6b). TAG is indicated by an *arrow*. **b** TAG quantities in mature endosperm and embryo of wild-type and AtG6b were determined by gas chromatography. *Error bars* indicate the standard deviation and *n* = 3. **P < 0.01 by Student’s *t*-test. **c** Percentage of various fatty acid compositions in the whole seed, endosperm, and embryo, respectively. *Error bars* indicate the standard deviation and n = 3

In Arabidopsis seed, oil is synthesized in both endosperm and embryo. In order to measure their TAG levels, embryo and endosperm/seed coat of mature seeds were separated as describe by Penfield et al. [22, 23] (Fig. 7a), and then lipid assays were further conducted. Based on thin layer chromatography (TLC) results, we determined that the TAG spots from embryo and endosperm of AtG6b seeds were larger than the corresponding spots from the ones of Col-0 seeds (Fig. 7a). Furthermore, quantitative analyses by gas chromatography (GC) revealed that the TAG contents of the embryo (36.29% ± 0.55) and endosperm (8.44% ± 0.78) from AtG6b seeds were significantly higher than the ones (29.55% ± 0.39, 5.15% ± 0.51) from Col-0 seeds (Fig. 7b). Different from TAG contents, the ratios of different fatty acid components from the whole seed, embryo, and endosperm were almost the same between AtG6b and Col-0 seeds (Fig. 7c).

### T-6b represses mucilage biosynthesis

**Fig. 8:**
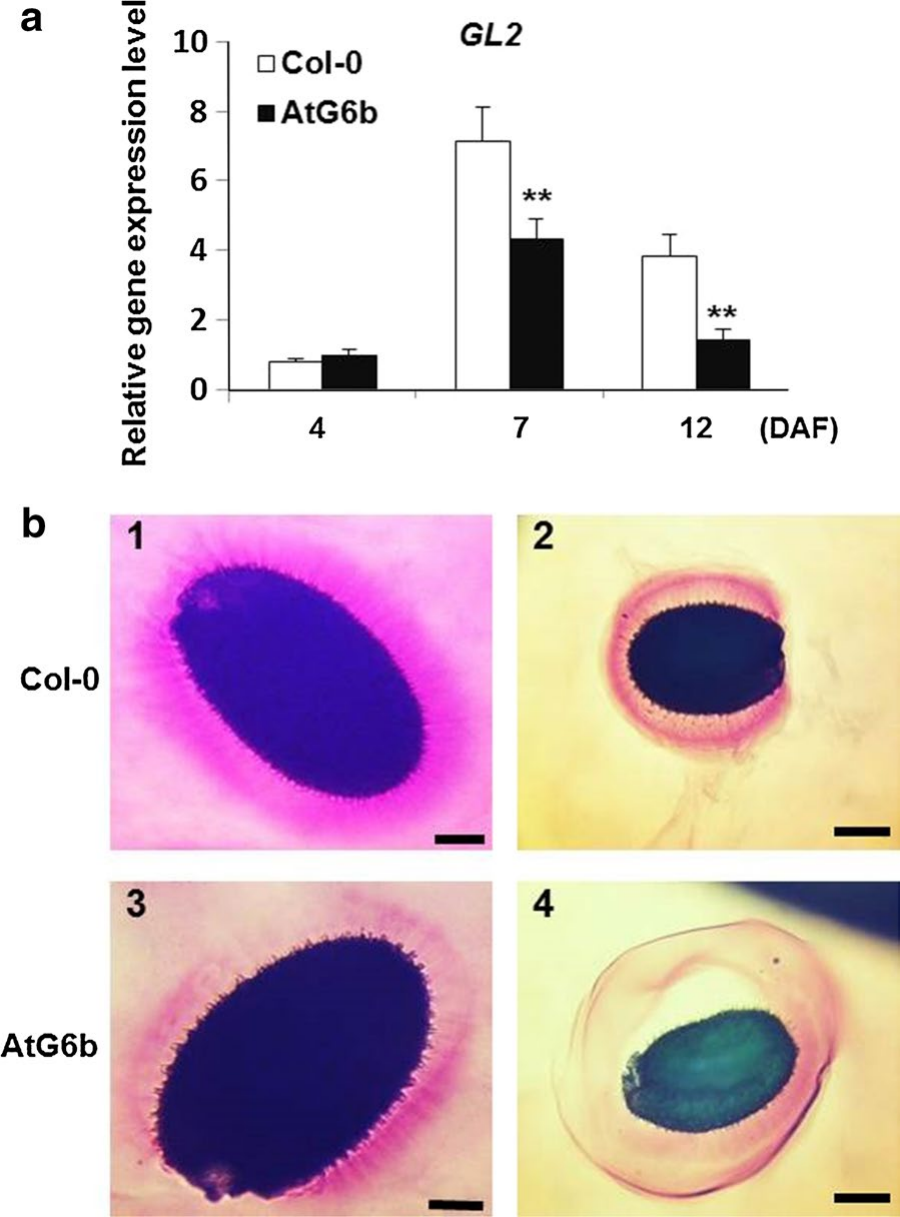
Effects of T-6b expressed in the endosperm on seed mucilage. **a** Expression levels of *GLABRA2* in different developing stage siliques of Col-0 and AtG6b. *Error bars* indicate the standard deviation and n = 3. **P < 0.01 by Student’s *t-*test. **b** Observation of seed mucilage released from wild-type (*1* and *2*) and AtG6b (*3* and *4*). (*1* and *3*) dried seeds were stained directly. (*2* and *4*) dried seeds were stained after imbibition for 1 h in water. *Bars* in *1* and *3* = 100 µm, *2* and *4* = 200 µm

It is unclear whether T-6b affects the distribution of carbon to the mucilage surrounding the seed coat. Therefore, we investigated the expression levels of *GLABRA2*, which is responsible for mucilage synthesis [25]. This gene was expressed at lower levels in AtG6b seeds than in the wild-type controls (Fig. 8a). Additionally, we stained the seed mucilage with ruthenium red, and observed that mucilage biomass was considerably lower for AtG6b seeds than for Col-0 seeds based on the intensity of the pink stain (Fig. 8b1, 3). Furthermore, the integrity of the mucilage layer surrounding the AtG6b testa was compromised (Fig. 8b2, 4). Because of the negative relationship between mucilage abundance on the seed coat surface and TAGs in the seed [24–26], we believe that the role of T-6b in reducing the mucilage abundance is beneficial to increase seed oil content.

## Discussion

Among the *6b* T-DNA genes from all *A. tumefaciens* strains, T-*6b* is the most oncogenic. The ectopic expression of *6b* results in the development of the enation syndrome in vegetative tissues [5–7]. Our investigation revealed that the ectopic expression of T-*6b* in reproductive tissues (e.g., endosperm) increases seed biomass without compromising the development of vegetative tissues (Fig. 1b). In other words, T-6b induces the enation syndrome in vegetative tissues, but only increases the biomass of reproductive organs. It is likely that T-6b up-regulates the expression of genes responsible for endosperm development, including *IKU1*, *IKU2*, and *MINI3* (Fig. 2), which increases the size of the ovaries to produce relatively large sinks. Therefore, the T-*6b* gene may be useful for increasing rapeseed crop yields.

T-6b activities increase leaf glucose, sucrose, and starch contents by regulating carbohydrate metabolism [7–10]. The expression of T-*6b* in the endosperm of *A. thaliana* plants increased glucose contents, but decreased the abundance of starch and sucrose in developing or mature seeds (Figs. 3, 4 and 5). The decrease in starch and sucrose contents was accompanied by down-regulated expression of the starch synthesis-related genes *SBE2.1* and *APS1* (Fig. 3a) and up-regulated expression of *Sus2* and *A/N*-*InvA*, which encode sucrose-degrading proteins (Fig. 5). As is known, WRI1 controlled by the B3 family is crucial for the synthesis of fatty acid [55, 56]. The up-regulated expression of *WRI1* suggests that T-6b or 6b-induced mobile enation factors induce the glycolytic pathway in the endosperm and embryo, leading to increased availability of acetyl-CoA for the synthesis of fatty acids in the endosperm and embryo. The different effects of T-6b on carbohydrate metabolism in vegetative and reproductive organs imply that the T-6b functions influencing sugar metabolism are more complicated than expected. Additionally, the T-6b molecular activities that regulate glucose contents are possibly the same in vegetative and reproductive organs, in contrast to the mechanisms mediating sucrose and starch metabolism. These possibilities warrant further study.

In wild-type *A. thaliana* seeds, carbon is primarily stored in lipids [51]. The endosperm begins to form 12 h after fertilization, while it starts to degrade at 12 DAF, which correlates with the timing of starch synthesis and degradation. Cotyledons then form up until 16 DAF, by which time the seeds have completed their development [15]. The expression of T-*6b* in the developing endosperm results in significantly up-regulated *WRI1* and *DGAT1* transcription and ultimately increases the seed TAG content (Figs. 6, 7). At the same time, T-6b decreases the mucilage biomass by repressing *GLABRA2* expression (Fig. 8). Therefore, T-*6b* expression in the endosperm leads to enhanced distribution of photosynthesis assimilation products to form TAGs, which function as the final storage products. The ability of T-6b to increase the allocation of carbon to TAGs in seeds may be useful for increasing crop yield.

Finally, our results expand our understanding of T-6b functions. To the best of our knowledge, this is the first study to confirm that unlike the effects of T-*6b* expression in vegetative tissues, the expression of this gene in reproductive organs leads to increased seed biomass and greater partitioning of photosynthates to TAGs without causing the enation syndrome. Our findings may form the basis of future studies aimed at improving oilseed crop yields.

## Conclusion

A novel function of T-6b gene was found in this study. Previous researches showed that ectopic expression of *T*-*6b* gene in vegetative organ led to enation syndrome and accumulation of soluble sugar around the gene expression loci. After letting the gene specifically express in reproductive organ seed, the T-6b not only caused no enation syndrome of seed, but also reduced the soluble sugar content, while increasing mature seed biomass and oil content. Our findings suggest that T-6b may be very useful for increasing oil production in biodiesel plants.

## Abbreviations

TAG: triacylglycerol
DAG: diacylglycerol
FFA: free fatty acids
PL: phospholipids
SBE2.1: starch branching enzyme 2.1
APS1: ADP glucose pyrophosphorylase 1
Sus2: sucrose synthase 2
A/N-InvA: alkaline/neutral invertase A
DGAT1: acyl-CoA:diacylglycerol acyltransferase 1
